# Counting the Cost of Diabetes in the Solomon Islands and Nauru

**DOI:** 10.1371/journal.pone.0145603

**Published:** 2015-12-23

**Authors:** Si Thu Win Tin, George Iro, Eva Gadabu, Ruth Colagiuri

**Affiliations:** 1 The Boden Institute of Obesity, Nutrition, Exercise & Eating Disorders, The University of Sydney, New South Wales, Australia; 2 National Diabetes Centre, Ministry of Health and Medical Services, Honiara, The Solomon Islands; 3 National Diabetes Centre, Ministry of Health and Medical Services, Republic of Nauru; 4 Menzies Centre for Health Policy, The University of Sydney, New South Wales, Australia; National Cancer Institute, UNITED STATES

## Abstract

**Aim:**

To determine the costs associated with diabetes to governments, people with diabetes and their carers, and its impact on quality of life in two Pacific Island countries—the Solomon Islands and Nauru.

**Materials and Methods:**

This cross-sectional cost of illness study was conducted on 330 people with type 2 diabetes (197 from the Solomon Islands and 133 from Nauru) using a structured cost of illness survey questionnaire adapted from the Australian DiabCo$t study. Quality of life was measured by the EQ-5D Visual Analogue Scale.

**Results:**

There were 330 respondents (50% female; mean duration of diabetes 10.9 years; mean age 52.6 years). The estimated annual national cost of diabetes incurred by the Solomon Islands government was AUD12.8 million (AUD281 per person/year) and by Nauru government was AUD1.2 million (AUD747 per person/year). The major contribution to the government costs was inpatient services cost (71% in the Solomon Islands and 83% in Nauru). Annual expenditure for diabetes was approximately 20% of the governments’ annual health care expenditure. Considerable absenteeism and retirement from work due to diabetes was found.

**Conclusions:**

This study found substantial public and personal costs associated with diabetes. The findings provide objective data on which health policy, funding and planning decisions about the prevention and control of diabetes in the Solomon Islands and Nauru can be reliably based and subsequently evaluated.

## Introduction

Diabetes imposes a substantial economic burden on national health systems globally [1,2]. For example, it is predicted that global health expenditure on diabetes will increase from USD376 billion in 2010 to some USD490 billion by 2030 [1]. Given the predicted increase in global diabetes prevalence from 382 million in 2013 to 592 million by 2035 [3], this raises serious concerns about the sustainability of the health systems especially in low and middle income countries (LMICS).

The cost of diabetes to individuals and families is also high. People with diabetes use more health services and spend approximately 2–3 times more on health care than people without diabetes [4,5]. In addition to health care expenditure, diabetes can reduce household income through lost employment due to illness and early retirement [6]. In countries with no social protection, it can also be a significant contributor to financial hardship and poverty [6,7]. Diabetes also incurs considerable intangible costs including reduced quality of life [5,8] and ranks among the top 10 causes of disability worldwide [6]. So it is not surprising that perhaps the greatest financial concerns about diabetes relate to its macroeconomic impact in the form of lost national productivity.

In the Western Pacific Region, Pacific Islands countries (PICs) have among the highest prevalence of diabetes in the world [2,3] and a high prevalence of diabetes complications [9,10]. Although there are some technical reports available on the economic burden of non-communicable diseases (NCDs) in the PICs [11,12] and a peer-reviewed study examining the cost of diabetes in Vanuatu [13] as part of the baseline assessment for a diabetes capacity building project [14], evidence quantifying the economic costs of diabetes is very limited. Improved understanding of the economic and social burden of diabetes in PICs could help to inform and motivate policymakers in each country, and international donors providing assistance for both countries to invest more in the prevention and control of diabetes. Therefore, we aimed to measure the costs associated with diabetes to governments, people with diabetes and their carers, and its impact on quality of life of people with diabetes in the Solomon Islands and Nauru.

## Materials and Methods

This cross-sectional study was conducted on 330 people with known type 2 diabetes (197 from the Solomon Islands and 133 from Nauru) to assess diabetes related cost of illness and quality of life. The study was undertaken as one of a number of baseline assessments for an overarching diabetes capacity building project [9,15]. The study in Nauru was conducted in 2007 and in the Solomon Islands in 2011.

A structured cost of illness survey questionnaire adapted from the Australian DiabCo$t study [16] was administered by in-country diabetes project staff. The questionnaire sought information on demographics; diabetes treatment—prescription medications, non-prescription medications and special food; health service utilisation—outpatient visits, inpatient stays and transport to attend health services; and its impact on quality of life. If respondents nominated a carer, a second section of the questionnaire assessing the cost associated with caring for a person with diabetes was administered to the nominated carer.

The distributions of total general population [17,18] and study population for both countries were compared. The number of hospital outpatient and inpatient services visited by the respondents and the cost of diabetes incurred for the three months preceding the study was assessed and multiplied by four to represent the annual number of visits and annual costs incurred. Health care costs for outpatient and inpatient services, and prescription medications were fully supported by governments of both countries ie no co-payment required by individuals and therefore these costs were reported as cost incurred by governments. The cost of non-prescription medications, and non-health care costs including transport to health care centres and purchase of special diabetic food were reported as cost incurred by individuals ie out-of-pocket expenses. Medication costs were sourced from the respective Ministry of Health and Medical Services (MHMS) pharmacies by the in-country project staff and estimation of costs was based on the medications and dosage received by the respondents at the time of the study. There was only one pharmacy in each country. Costing estimates for each outpatient clinic visit and per night inpatient stays were provided by the respective MHMS.

The national cost of diabetes in the Solomon Islands in 2011 among people aged 20–79 was calculated by multiplying the estimated prevalence of diabetes in 2011 [19] and the number of people aged 20–79 in 2011 [20] by the annual cost per person. The national cost of diabetes in Nauru in 2007 among people aged 20–79 was calculated by multiplying the estimated prevalence of diabetes in 2007 [21] and the number of people aged 20–79 in 2007 [20] by the average annual cost per person. The average exchange rate in 2011 was used to convert Solomon Islands Dollar (SBD) to Australian dollar (AUD) (7.9SBD = 1AUD) [22]. Nauru uses AUD as its official currency. The cost of diabetes in Nauru was inflated to 2011 cost using a web-based cost converter version 1.4 [23,24], which inflates for years and country, to align with the cost of diabetes reported in the Solomon Islands in 2011.

The data were compiled and analysed using IBM SPSS statistical package version 21 and Microsoft Office Excel. Quality of life was measured by administration of the EQ-5D questionnaire and analysed using the EuroQol EQ-5D user guide [25]. Where relevant, data are reported as mean ± SEM, minimum, maximum, median, number and percentage.

This study was conducted with the approval of the Human Research Ethics Committee, the University of Sydney and a Memorandum of Understanding between the researchers and the Ministries of Health in the Solomon Islands and Nauru. The participants provided written informed consent to participate in this study.

## Results

### Demographics

Table 1 shows the demographic details of the 197 people with type 2 diabetes (mean age 54.1 years, mean age at diagnosis 45.4 years, and mean duration of diabetes 8.7 years) in the Solomon Islands and 133 people with type 2 diabetes (mean age 50.6 years, mean age at diagnosis 36.4 years, and mean duration of diabetes 14.2 years) in Nauru. The percentage of respondents treated with insulin was very low in both countries (average 6.7%). Overall, 61.2% respondents are not in paid employment.

**Table 1:** Demographic details of people with diabetes.

	Solomon Islands	Nauru
	(N = 197)	(N = 133)
**Mean age** (years)	54.1±0.7	50.6±1.2
*Overall mean = 52*.*6*±0.6 *years*	(Min 22 –Max 84)	(min 20 –Max 81)
**Gender**		
- Male	52.3%	46.6%
- Female	47.7%	53.4%
*Overall male = 50%*, *female = 50%*		
**Mean age at diagnosis** (years)	45.4±0.7	36.4±1.1
*Overall mean = 41*.*8*±0.7 *years*		
**Mean duration of diabetes** (years)	8.7±0.5	14.2±1.0
*Overall mean = 10*.*9*±0.5 *years*	Median 6 (Min 1 –Max 40)	Median 13 (Min 1 –Max 55)
**Current smokers**	7.1%	33.8%
*Overall smokers = 17*.*9%*		
**Employment**		
- Full-time paid employment	27.4%	46.6%
- Part-time paid employment	3.6%	3.8%
- Not in paid employment	69.0%	49.6%
*Overall full-time paid employment = 35*.*2%*, *part-time paid*		
*employment = 3*.*6%*, *not in paid employment = 61*.*2%*		
**Diabetes treatment**		
- Diet alone	16.8%	44.4%
- Oral anti-diabetic tablets	76.6%	48.8%
- Insulin	6.6%	6.8%
*Overall diet alone = 27*.*9%*, *oral anti-diabetic tablets* =		
*65*.*4%*, *insulin = 6*.*7%*		

Data are shown as mean±SEM or minimum/maximum or median or percentage.

**Fig 1:**
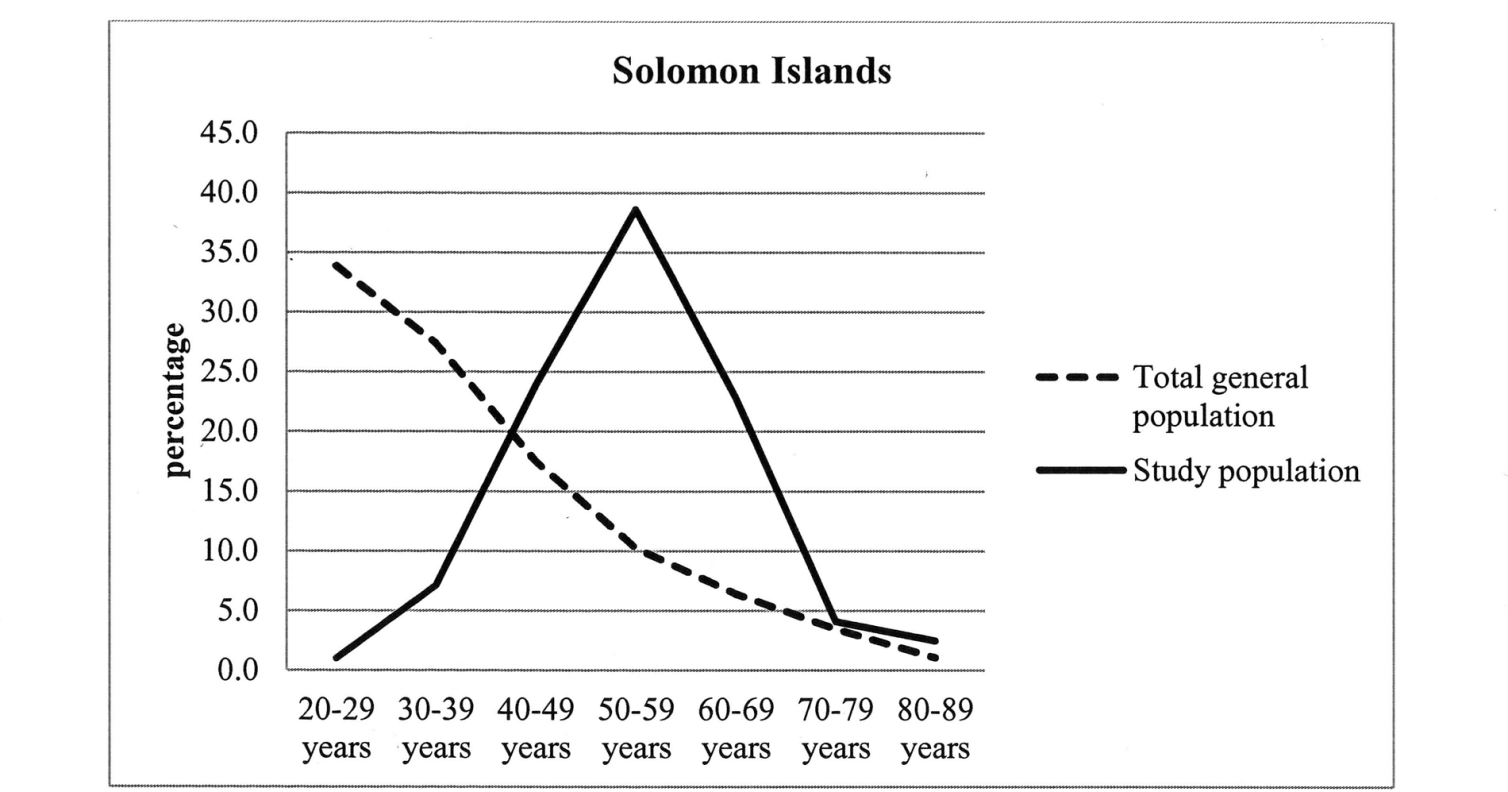
Distribution of total general population and study population in the Solomon Islands.

**Fig 2:**
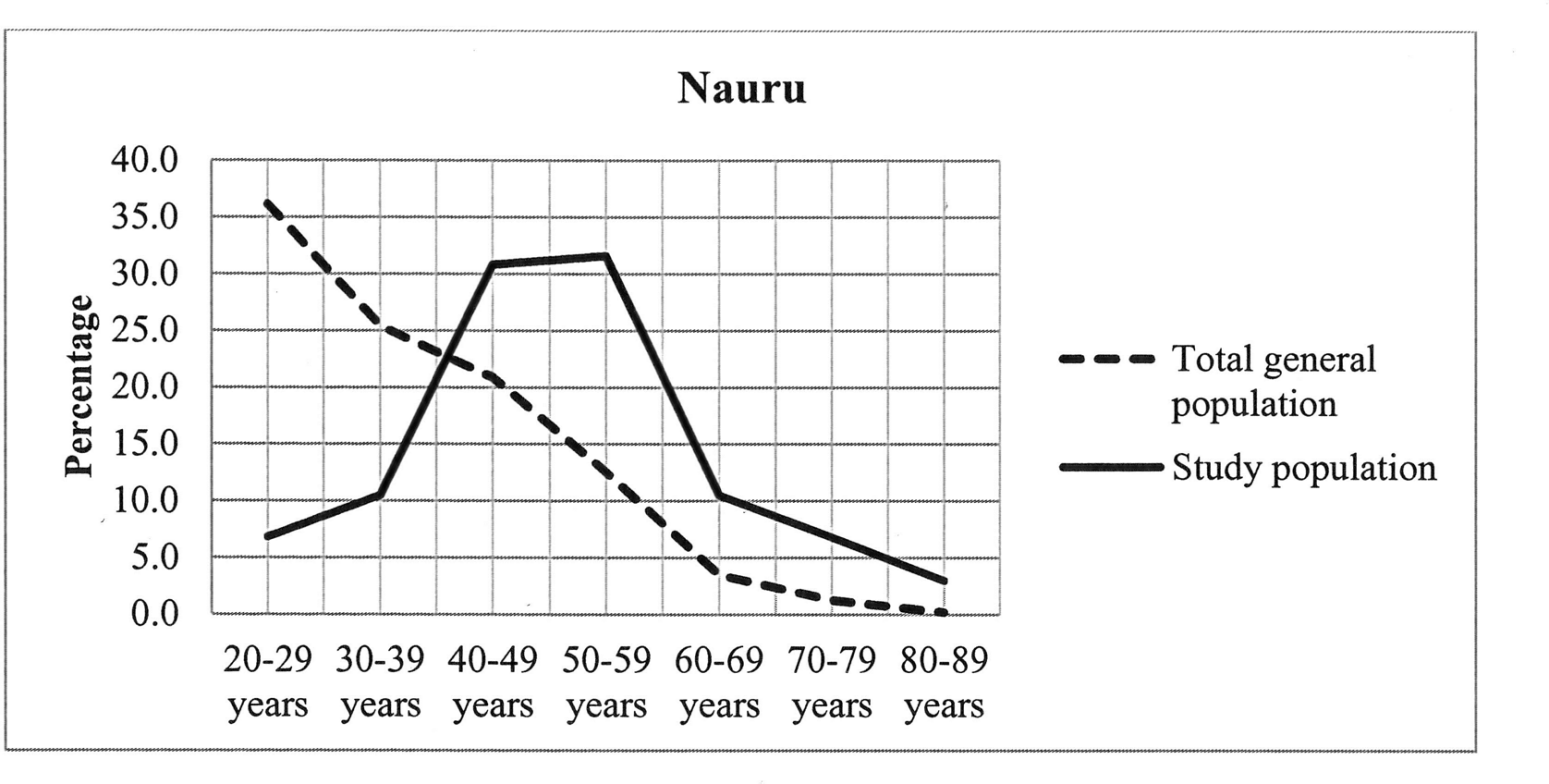
Distribution of total general population and study population in Nauru.

The distribution of total general population and our study population in different age groups in the Solomon Islands and Nauru were presented in Figs 1 and 2 respectively. The highest percentages of respondents (38.6% in the Solomon Islands and 31.6% in Nauru) in our study population were found aged between 50 and 59 years.

### Government costs

Table 2 shows estimated cost of diabetes incurred by governments ie the cost of outpatient and inpatient services and prescription medications.

For the Solomon Islands, there were 1,084 visits to the hospital outpatient clinic/diabetes centre and 88 visits to the hospital emergency department totalling 1,172 (average 6 visits/ person/ year) outpatient visits per year reported. For Nauru, there were 1,184 visits to the hospital outpatient clinic/diabetes centre and 68 visits to the hospital emergency department totalling 1,252 (average 9 visits/ person/ year) outpatient visits per year reported. Overall 532 days (average 3 days/ person/ year) and 972 days (average 7 days/ person/ year) overnight hospital stays per year were reported from the Solomon Islands and Nauru respectively.

**Table 2:** Estimated cost of diabetes incurred by governments.

	Solomon Islands				Nauru	
	Annual cost		Annual		Annual cost	Annual
	per person		national cost		per person	national cost
	(N = 197)		(N = 45,465)*		(N = 133)	(N = 1,571)*
			(cost in 1000s)			(cost in 1000s)
	SBD	AUD	SBD	AUD	AUD	AUD
**Outpatient** **	**$119**	**$15±2**	**$5,410**	**$685±81**	**$28±3**	**$44±5**
*Solomon Islands = average 6±0*.*7 visits/person/year*						
*Nauru = average 9±1*.*1 visits/person/year*						
**Inpatient** ***	**$1,620**	**$205±61**	**$73,667**	**$9,325±2783**	**$621±147**	**$975±230**
*Solomon Islands = average 6±2*.*4 days/person/year*						
*Nauru = average 9±2*.*1 days/person/year*
**Prescription medications** ****						
▪ Oral hypoglycaemic	$205	$26	$9,314	$1,179	$29	$46,
▪ Insulin	$144	$18	$6,569	$832	$27	$42
▪ Antihypertensive	$94	$12	$4,291	$543	$18	$28
▪ Antibiotic	$16	$2	$706	$89	$21	$31
▪ Pain relief / anti-inflammatory	$20	$3	$918	$116	$2	$4
**Total cost for prescription medications**	**$479**	**$61±1.9**	**$25,215**	**$3,198±86**	**$98±2.6**	**$153±4**
**Overall cost incurred by governments**	**$2,218**	**$281±62**	**$100,874**	**$12,769±2805®**	**$747±148**	**$1,173±231®**

*People with diabetes aged 20–79 years

** *Solomon Islands = number of persons reported 91(46*.*2%) with minimum cost AUD10 /year and maximum cost AUD243/year*. *Nauru = number of persons reported 81 (60*.*9%) with minimum cost AUD12/year and maximum cost AUD288/year*.

*** *Solomon Islands = number of persons reported 15(7*.*5%) with minimum cost AUD303/year and maximum cost AUD6075/year*. *Nauru = number of persons reported 20 (15*.*0%) with minimum cost AUD680/year and maximum cost AUD9520/year*.

**** *Solomon Islands = number of persons reported on oral-anti-diabetic tablets 151 (76*.*6%); insulin 13 (6*.*6%); antihypertensive 44 (22*.*3%); antibiotics 4(2*.*0%); pain relief/anti-inflammatory 4 (2%)*. *Nauru = number of persons reported on oral-anti-diabetic tablets 65 (48*.*9%); insulin 9(6*.*8%); antihypertensive 31 (23*.*3%); antibiotics 12(9*.*0%); pain relief/anti-inflammatory 3(6*.*8%)*.

*®* Approximately 20% of the respective government’s annual health care expenditure

SBD = Solomon Islands Dollar; AUD = Australian Dollar

In 2011, 291,444 adults in the Solomon Islands were aged between 20–79 years [17]. Of these, 15.6% or 45,465 were estimated to have diabetes [16]. In 2007, 5,118 adults in Nauru were aged between 20–79 years [18] nationally. Of these, 30.7% or 1,571 were estimated to have diabetes [19]. The estimated annual national cost of diabetes incurred by the Solomon Islands government was AUD12.8 million (SBD100.9 million) and by the Nauru government was AUD1.2 million (Table 2).

The overall breakdown of the contribution to costs for the Solomon Islands government was 5% for outpatients, 71% for inpatients and 24% for prescription medications. For the Nauruan government the costs were 4% for outpatients, 83% for inpatients and 13% for prescription medications. The overall breakdown of contributions to the cost of prescription medications in the Solomon Islands was 37% for oral hypoglycaemic, 34% for insulin, 23% for antihypertensives, and 3% each for antibiotics and pain relief or anti-inflammatories. The overall breakdown of the contribution to prescription medications in Nauru was 30% for oral hypoglycaemics, 28% for insulin, 19% for antihypertensives, and 21% for antibiotics and 2% for pain relief or anti-inflammatories.

### Individual costs

Costs incurred by people with diabetes (non-prescription medications, special diabetic food and transport to health care centres) were assessed. The average annual cost per person was AUD99 (SBD780) in the Solomon Islands and AUD110 in Nauru. The estimated annual national cost incurred by individuals was approximately AUD4.5 million (SBD35.5 million) in the Solomon Islands and AUD0.17 million in Nauru (Table 3).

**Table 3:** Estimated cost of diabetes incurred by people with diabetes.

	Solomon Islands			Nauru		
	Annual cost		Annual		Annual cost	Annual
	per person		national cost		per person	national cost
	(N = 197)		(N = 45,465)*		(N = 133)	(N = 1,571)*
			(cost in 1000s)			(cost in 1000s)
	SBD	AUD	SBD	AUD	AUD	AUD
Non prescriptive medications cost**	$81	$10±2.8	$3,683	$466±128	$33±6	$52±8
Special diabetic food cost***	$384	$49±7	$17,459	$2,210±334	$41±12	$64±19
Transport cost****	$315	$40±10	$14,322	$1,813±457	$36±5.9	$57±9
**Total cost incurred by individuals**	**$780**	**$99±15**	**$35,463**	**$4,489±668**	**$110±15**	**$173±23**

*People with diabetes aged 20–79 years

** *Solomon Islands = number of persons reported 11(5*.*6%) with minimum cost AUD5/year and maximum cost AUD202/year*. *Nauru = number of persons reported 69 (52*.*0%) with minimum cost AUD10/year and maximum cost AUD240/year*.

*** Solomon Islands = number of persons reported 80(40.*6%) with minimum cost AUD10/year and maximum cost AUD911/year*. *Nauru = number of persons reported 13 (9*.*8%) with minimum cost AUD24/year and maximum cost AUD800/year*.

****Solomon Islands = number of persons reported 43(21.*8%) with minimum cost AUD10/year and maximum cost AUD253/year*. *Nauru = number of persons reported 51 (38*.*3%) with minimum cost AUD16/year and maximum cost AUD200/year*.

Of the respondents who were in paid employment at the time of study, overall 31.1% of respondents (37.7% from the Solomon Islands and 25.4% from Nauru) had taken time off work of approximately 10 days per person/year due to diabetes. Overall 8.8% of respondents (7.1% from the Solomon Islands and 11.3% from Nauru) were retired due to diabetes.

### Quality of life

With regard to quality of life, the EQ-5D average visual analogue scale (VAS) reported was 73% (range 22–100) in the Solomon Islands and 77% (range 25–100) in Nauru. When results from both countries were combined, the overall average VAS reported was 75% (range 22–100). Table 4 shows self-reported EQ-5D score.

**Table 4:** Self-reported EQ-5D score.

	Solomon Islands	Nauru
	n (%)	n (%)
	(N = 197)	(N = 133)
**Mobility**		
- No problems in walking about	129 (65.5%)	88 (66.2%)
- Some problems in walking about	68 (34.5%)	42 (31.5%)
- Confined to bed	0 (0.0%)	3 (2.3%)
**Self-care**		
- No problems with self-care	168 (85.3%)	115 (86.5%)
- Some problems washing or dressing myself	25 (12.7%)	14 (10.5%)
- Unable to wash or dress myself	4 (2.0%)	4 (3.0%)
**Usual activities** (eg work, study, housework, family or leisure activities)		
- No problems with performing my usual activities	141(71.6%)	96 (72.2%)
- Some problems with performing my usual activities	42 (21.3%)	27 (20.3%)
- Unable to perform usual activities	14 (7.1%)	10 (7.5%)
**Pain/discomfort**		
- No pain or discomfort	54 (27.4%)	74 (55.6%)
- A small amount of pain or discomfort	130 (66.0%)	52 (39.1%)
- A lot of pain or discomfort	13 (6.6%)	7 (5.3%)
**Anxiety/depression**		
- Not anxious or depressed	71 (36.0%)	88 (66.2%)
- A little anxious or depressed	115 (58.4%)	37 (27.8%)
- Very anxious or depressed	11 (5.6%)	8 (6.0%)

### Diabetes carers

Of the 330 people with diabetes, 93 (28%) [77(39%) from the Solomon Islands and 16 (12%) from Nauru] nominated a carer. Table 5 shows the demographic details of the 93 carers (overall mean age 55 years and female 71%). Overall 63.4% were not in paid employment and 7.5% had retired to look after person with diabetes. Over 90% of carers were a family member who lived with person with diabetes and did not receive any carer payment. The average time spent directly caring for people with diabetes was 3 days and 5 days per week (159 and 278 days per year) among carers from the Solomon Islands and Nauru respectively. The care for people with diabetes comprised preparing meals, transporting to their doctors, administering medications, assisting daily work particularly for people with complications.

**Table 5:** Demographic details of diabetes carers.

	Solomon Islands	Nauru
	(n = 77)	(n = 16)
Mean age (years)	44.6±1.5	46.8±4.5
*Overall mean = 45*±1.5 *years*	(Min 20 –Max 69)	(Min 21 –Max76)
Gender		
- Male	32.5%	12.5%
- Female	67.5%	87.5%
*Overall male = 29%*, *female = 71%*		
Employment		
- Full-time paid employment	33.8%	25.0%
- Part-time paid employment	3.9%	6.3%
- Not in paid employment	62.3%	68.7%
*employment = 4*.*3%*, *not in paid employment = 63*.*4%*		
Retired to look after person with diabetes	6.5%	12.5%
*Overall = 7*.*5%*		
Carer with diabetes	6.5%	6.3%
*Overall = 6*.*5%*		
Relationship with person with diabetes		
- Husband/wife/partner	58.4%	62.5%
- Another family member	40.3%	37.5%
- Friend or neighbour	1.3%	0.0%
*Overall husband/wife/partner = 59*.*1%*, *another family member =*		
*39*.*8%*, *friend or neighbour = 1*.*1%*		
Live with person with diabetes	94.8%	93.8%
*Overall = 94*.*6%*		

Costs incurred by carers (non-prescription medications, special diabetic food and transport to health care centres) spent caring for the person with diabetes were assessed. The average annual cost per carer was AUD439 (SBD3,471) in the Solomon Islands and AUD492 in Nauru (Table 5).

Of the 45,465 estimated people with diabetes aged 20–79 years in the Solomon Islands, it was estimated that 17,771 (39%) had carers and in Nauru 182 (12%) of the 1,517 estimated people with diabetes aged 20–79 had carers. The national cost of diabetes incurred by carers was calculated by multiplying the estimated number of diabetes carers by the annual cost per carer. The estimated annual national cost of diabetes incurred by carers in the Solomon Islands was approximately AUD7.8 million (SBD61.7 million) and by carers in Nauru was approximately AUD0.9 million (Table 6).

**Table 6:** Estimated cost of diabetes incurred by carers.

	Solomon Islands			Nauru		
	Annual		Annual		Annual	Annual
	cost per person		national cost		cost per person	national cost
	(N = 77)		(N = 17,771)*		(N = 16)	(N = 182)*
			(cost in 1000s)			(cost in 1000s)
	SBD	AUD	SBD	AUD	AUD	AUD
Non prescriptive medications cost**	$596	$75±13	$10,591	$1,333±220	$176±40	$32±7
Special diabetic food cost***	$1,925	$244±26.4	$34,203	$4,336±471	$289±66	$53±11
Transport cost****	$950	$120±17	$16,880	$2,133±313	$27±9.7	$5±0.2
**Total cost incurred by individuals**	**$3,471**	**$439±35**	**$61,674**	**$7,802±624**	**$492±74**	**$90±13**

*People with diabetes aged 20–79 years

** *Solomon Islands = number of persons reported 30(39*.*0%) with minimum cost AUD6/year and maximum cost AUD303/year*. *Nauru = number of persons reported 9 (56*.*3%) with minimum cost AUD12/year and maximum cost AUD240/year*.

*** Solomon Islands = number of persons reported 67(87.*0%) with minimum cost AUD25/year and maximum cost AUD506/year*. *Nauru = number of persons reported 9 (56*.*3%) with minimum cost AUD16/year and maximum cost AUD400/year*.

****Solomon Islands = number of persons reported 53(68.*8%) with minimum cost AUD10/year and maximum cost AUD354/year*. *Nauru = number of persons reported 6 (37*.*5%) with minimum cost AUD20/year and maximum cost AUD200/year*.

## Discussion and Conclusion

This study found a substantial cost associated with diabetes to governments, people with diabetes and their carers, as well as social problems associated with diabetes in PICs. This included considerable absenteeism and retirement from work due to diabetes.

Of the cost incurred by the governments, annual inpatient services accounted for the highest expenditure (71% in the Solomon Islands and 83% in Nauru). This is consistent with previous studies [13,26–28] and may be due to the high prevalence of diabetes complications previously reported in Nauru and the Solomon Islands [9]. Despite high prevalence of diabetes complications reported in these countries [9], our study found that the percentage of respondents treated with insulin was relatively low (approximately 6%). Our study did not investigate the reasons for this, however, factor may be the relative absence of other forms of management to improve diabetes control. Given the high rate of complications and low rate of insulin usage, there is an urgent need to review protocol and guidelines for treating people with diabetes to reduce complications in these PICs. It was also found that respondents in Nauru developed diabetes at a young age and have long duration of diabetes. This highlights the need to invest more and strengthen prevention and control of diabetes particularly in Nauru.

Although the direct health care costs were relatively low when compared with the costs reported in other countries [8,27,29–31], costs incurred by governments were substantial for these small island nations. For example, the annual expenditure on diabetes in the Solomon Islands and Nauru found in this study was approximately 20% of the governments’ annual health care expenditure [32–35] which is higher than the average health spending (10.8%) on diabetes worldwide [2]. Most PICs have a traditional, informal system of ‘universal health care coverage; in that patients do not pay for visits to public health facilities or health care providers, or medications thus the cost of diabetes care to governments versus patients is high. This highlights the disproportionate cost of diabetes by the governments in resource constrained PICs many of which already suffer severe economic hardship and development challenges.

The unemployment rate in our study population (69.0% in the Solomon Islands and 49.6% in Nauru) was much higher than that of the whole population (39.8% in the Solomon Islands and 23.0% in Nauru [36–38]) in both countries. This could be a result of high complications rates among people with diabetes in both countries. In addition, given the high proportion of absenteeism (37.7% in the Solomon Islands and 25.4% in Nauru) and retirement from work (7.1% in the Solomon Islands and 11.3% in Nauru) due to illness resulting from diabetes, out-of-pocket expenses incurred by people with diabetes is of considerable concern. Possibly as a result of the lack of national social protection, rather than paying their carers, some respondents appeared to be financially dependent on their carers. Since most carers were also not in paid employment and some had retired in order to look after the person with diabetes, the financial cost and social burden faced by carers—usually family members—in PICs is a potential cause of poverty which may even translate into inter-generational financial hardship and poverty. With high unemployment and relatively low income per capita in PICs compared with developed countries [36–38], any out-of-pocket expense incurs a significant financial burden for Pacific people.

The long mean duration of diabetes found in our study, the poor glycaemic control and the high prevalence of diabetes complications reported in both countries [9] could explain their high number of outpatient visits and inpatient stays. Consequently, the average annual costs of diabetes per person incurred by government, individuals and carers in both countries were substantial. Evidence from previous studies has also shown that the cost of diabetes is higher in people with diabetes complications [16,28,39–43].

People in the Pacific have strong family support which is a well recognized Pacific cultural trait. For example, of the 93 carers we surveyed, over 90% were family members and lived with the person with diabetes. Thus, the relatively low impact of diabetes on quality of life (VAS) reported in this study despite high rates of diabetes complications [9] may result from cultural factors such as strong family support and/or low expectations of wellness and quality of life.

Although our study has identified major costs related to diabetes in PICs, it has certain limitations. The costs of non prescription medications, special diabetic food and transport as well as the number of hospital stays and diabetes centre visits were self-reported and relied solely on respondents re-call. Consequently the estimated costs may not precisely reflect the actual costs. Further, it is possible that out-of-pocket expenses incurred by both people with diabetes and carers could have been overestimated as respondents may have reported food and transport costs not specifically related to diabetes. Given the small sample size particularly the carers, it is difficult to conclude that this is a representative sample in both countries. National cost estimates were based on an age range of 20–79 years because the prevalence of diabetes was available only for age rage 20–79 years in both countries. Five respondents (2.5%) from the Solomon Islands and 4 respondents (3%) from Nauru in our study population were over 79 years. However, such a small proportion of respondents above the age range would be unlikely to impact significantly on the results. For example, if patients above 79 years old were excluded in the cost calculation, the average annual cost per person difference was only AUD0.2 less in both countries and therefore there any difference in national cost estimates would be very small.

Despite these limitations the study has certain strengths. The survey questionnaire was adapted to ensure cultural appropriateness and administered by local staff who underwent specific training for the task. For accuracy, the cost estimations for prescription medications was based on the medications and dosage received by the respondents recorded in medical records. Calculating annual costs by extrapolating from data collection covering a three month period is acceptable and established practice [16,39]. Costs for each outpatient clinic visit and inpatient per night stay provided by the respective MHMS were almost similar and also closely aligned with the costs reported in other published literature [11–13]. The estimated population aged 20–79 used in this study [20] was fairly consistent with the national population census reported by the Bureau of Statistics in the Solomon Islands and Nauru [17,18].

In conclusion, this study adds important information to the pool of knowledge about diabetes in PICs. It also contributes to the international evidence on the cost of diabetes to governments, and people with diabetes and their carers. Given the increasing prevalence and impact of diabetes in LMICs, it provides a timely reminder to government in each country, and international donors providing assistance in both countries that urgent action is required to reduce the public and personal cost burden of diabetes in PICs. Comparable research is needed to precisely determine the cost burden of diabetes in other PICs, as are more detailed studies on cost effectiveness across the Pacific Islands. In the meantime, these findings particularly a substantial cost associated with diabetes, diagnosed at early age, considerable absenteeism and retirement from work due to diabetes provide objective data on which health policy, funding and planning decisions about the prevention and control of diabetes in the Solomon Islands and Nauru can be reliably based and subsequently evaluated.
